# A Cytomegalovirus (CMV) Case Study to Promote Interprofessional Learning (IPL) Between Audiology and Biomedical Science Students in Higher Education

**DOI:** 10.3389/bjbs.2023.11680

**Published:** 2023-11-29

**Authors:** Amreen Bashir, Ross Pallett, Karan Singh Rana, Saira Hussain

**Affiliations:** ^1^ School of Biosciences, College of Health and Life Sciences, Aston University, Birmingham, United Kingdom; ^2^ Department of Audiology, College of Health and Life Sciences, Aston University, Birmingham, United Kingdom

**Keywords:** interprofessional learning, audiology, biomedical science, higher education, patient care

## Abstract

Modern and effective patient care requires specialist healthcare professionals working together. Interprofessional learning (IPL) seeks to provide opportunities for different healthcare disciplines to learn with, from and about each other. This study focused on the delivery and evaluation of a cytomegalovirus (CMV) case study workshop to facilitate IPL between two Health and Care Professions Council (HCPC) regulated courses: Biomedical Science and Audiology. The 2 h online workshop consisted of 1) defining the roles, responsibilities and skills of the two healthcare professions, 2) the structure of the Biomedical Science and Audiology departments, 3) routes to HCPC registration, 4) core curriculum of both degree programmes and 5) interpreting interdisciplinary data related to a CMV patient case. The workshop was interactive, with the virtual learning environment promoting peer discussions and the use of online polling. Student responses were collected through an online questionnaire. A total of 108 respondents completed a post-event survey and Mann-Whitney *U* tests revealed there were no significant differences in the responses between the two student cohorts in response to each of the survey statements (*p* > 0.05). A total of 82.4% of students agreed that they need to know the role of other healthcare professionals for their future practice, whilst 84.2% agreed that the CMV case study was a good format to facilitate effective IPL. A total of 93.5% of respondents recognised the importance of both professions in diagnosing a patient with CMV. Thematic analysis identified four common themes, including appreciation of shared roles, recognition of similarities in registration pathways, working together to provide holistic patient care and the role of clinicians in the patient journey. This novel collaboration between Biomedical Science and Audiology facilitated effective IPL whilst meeting the interprofessional education HCPC requirements. Collaborative working is an essential component of delivering effective patient care and allied healthcare degrees need to provide opportunities within their curriculum to foster this. We hope this study encourages other higher education institutes to expand and develop their current IPL activities to include a broader spectrum of healthcare courses.

## Introduction

Modern healthcare is truly multidisciplinary in nature, requiring many highly skilled healthcare professionals working together to provide effective patient care [1]. Advancements in pathology over recent decades have helped to transform the diagnostic pathway, allowing healthcare professionals to better identify and treat conditions. Patients are presenting with increasingly complex conditions and co-morbidities which require multidisciplinary approaches to care. In many specialities such as oncology, endocrinology and palliative care, there are established teams that directly impact the patient pathway. However, other healthcare professionals often work and learn in silo, without developing an understanding of the roles of other healthcare professionals and how collaboration can improve patient outcomes [2].

Within the higher education setting, IPL between nursing and medical programmes is well established and interprofessional education (IPE) is an essential component of the curriculum of subjects allied to health. Numerous publications have shown how interactivity develops key skills, such as fostering collaboration, improving communication and shared decision making and improving one’s understanding of their role and responsibilities and the role and responsibilities of others [2–4]. Ultimately, IPE aims to improve the quality of care provided to patients by creating a more integrated and collaborative healthcare team.

### Recognition of IPL by the Health and Care Professions Council

The importance and value of IPL within the healthcare setting is well recognised, yet there has been a lag with regard to its implementation within undergraduate healthcare programmes such as Audiology and Biomedical Science. The Health Care and Professions Council (HCPC) accredit both aforementioned programmes and requires students enrolled on these courses to have opportunities to undertake IPL in order to “learn with, and from, professionals and learners in other relevant professions” [5]. As the importance of IPL is mandated by the HCPC it has been incorporated into both BMS and Audiology modules and programme specifications respectively. Students need to be able to “*explain, in an integrated manner, the importance of service users and the role of a multidisciplinary team in the delivery of effective patient care including inter-professional learning*”.

Biomedical Scientists (BMS) are involved in up to 95% of all clinical pathways [6] and yet this was traditionally under-recognised by both patients and other pathology service users [7]. The COVID-19 pandemic helped to put the profession of a Biomedical Scientist into the public eye, with individuals recognising the key role of laboratory scientists in the diagnosis, monitoring and treatment of patients. Whilst recent studies have evaluated the effectiveness of IPL between Biomedical Scientists and other healthcare programmes in a traditional face-to-face setting [8] and virtual learning environments [3], to our knowledge no other studies are exploring the value of IPL between a laboratory-based Biomedical Scientist and a patient facing Audiologist.

### The Value of IPL Between Biomedical Scientists and Audiologists

Audiologists play a central role in identifying, assessing, and treating hearing problems and balance disorders [9]. Whilst it may appear at first glance that there is little overlap between the two professions, there are many commonalities related to their role in providing effective patient care. Audiologists play a central role in diagnosing patients with hearing disorders, several of which are a result of bacterial and viral infections. One cause of hearing loss in children is cytomegalovirus (CMV), which can be transmitted to the foetus during pregnancy or delivery. It has been estimated that 1 in 200 babies are born with congenital CMV infection [10] and 10%–15% will have long-term health problems, including sensorineural hearing loss [11]. Testing for CMV viral infection and confirmation of its diagnosis is performed by a Biomedical Scientist, whereas the initial hearing screens and referral are performed by an Audiologist.

Thus, this study aimed to create and assess the effectiveness of an online IPL workshop involving final year Biomedical Science students and first-year Audiology students. The workshop focussed on a patient case study involving CMV and sought to improve awareness and understanding of both healthcare roles whilst highlighting the role of each profession in diagnosing and treating patients with a CMV infection.

## Materials and Methods

### Creation of a Virtual IPL Workshop

A 2 h online workshop was designed and co-delivered by academics between the Department of Audiology and the School of Biosciences at Aston University, United Kingdom. The preferred method of delivering the workshop was online in order to increase student engagement and to promote fruitful cross disciplinary interactions based on previous success. The workshop included fifteen first-year Audiology students and ninety-three final-year Biomedical Science students. In the context of IPL, year 1 Audiology students were designated to collaborate with final-year Biomedical Science students. This IPL activity was set to coincide with the curricular focus of Year 1 Audiology students, who were at a stage in their program where they were learning fundamental knowledge pertaining to screening principles and the underpinning principles of CMV assessment processes. Final-year Biomedical Science students were selected for this IPL activity as they complete clinical modules by this stage. Furthermore, as part of this module, students learn about NHS structure and specific HCPC requirements related to IPL and multidisciplinary working, prior to entering the graduate healthcare workforce.

**FIGURE 1:**
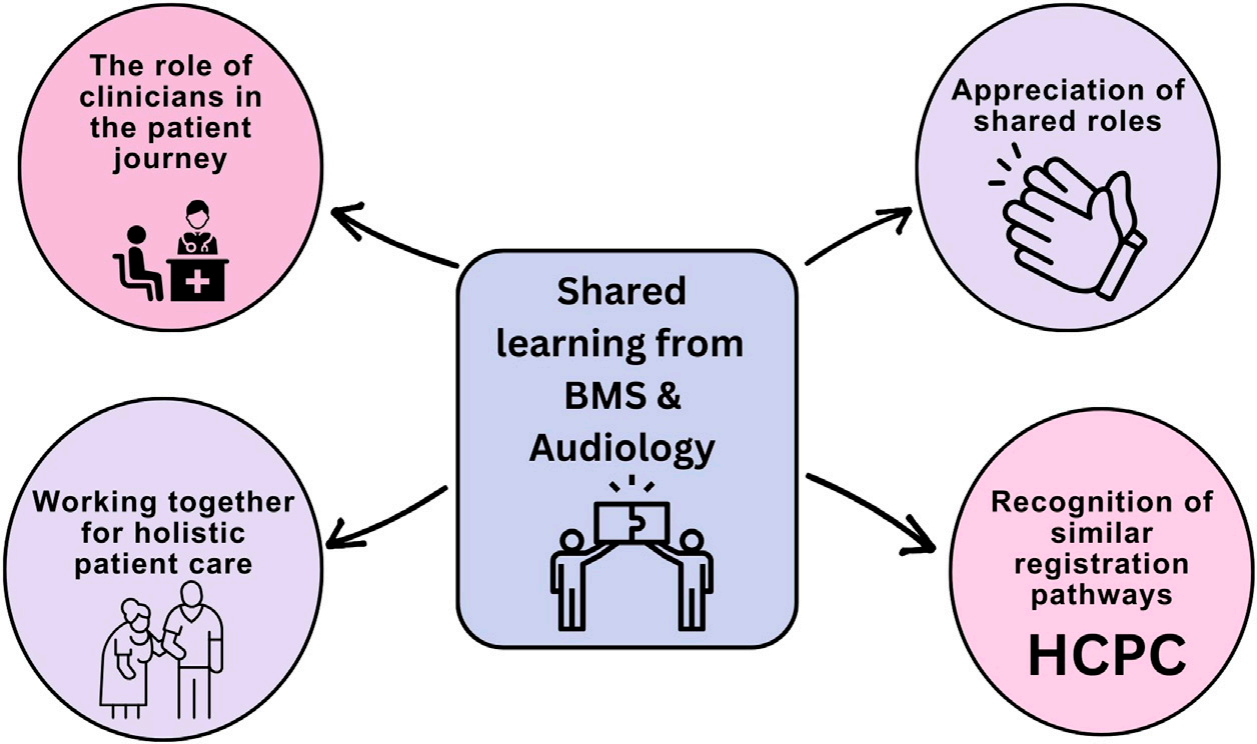
Steps involved in the design, delivery, and evaluation of the IPL workshop.

To promote IPE, the workshop included five key areas of delivery, which consisted of 1) the distinct roles and skills of an Audiologist and a Biomedical Scientist, 2) the structure of Audiology and Pathology departments, 3) routes to HCPC professional registration, 4) core components of an Audiology and Biomedical Science degree and 5) interpreting data relating to a CMV patient case study. The workshop was delivered using the virtual learning environment (VLE) Blackboard Collaborate platform (Blackboard, Washington DC). Using the breakout room function, students were assigned into seven mixed profession groups. The workshop was led by academic staff, with students participating through the use of the chat, audio and polling functions of the VLE. The IPL was co-created with academics from Biomedical Science and Audiology and the steps involved in the design, delivery and evaluation are detailed in Figure 1.

### Cytomegalovirus Case Study

Students were presented with a case study of a patient who contracted CMV during the last trimester of her pregnancy and was concerned that she had passed the virus on to her baby during delivery. The case provided a background to congenital CMV, its mode of transmission, virulence and the probability of the newborn baby developing sensorineural hearing loss. The case study then discussed CMV testing within the pathology laboratory, including the sample types required, the diagnostic tests performed in the virology and cytology laboratories, highlighting positive test results. The final part of the workshop introduced Audiology components, including hearing screening, the hearing pathway, sensorineural hearing loss and defining key terms such as “screening,” “sensitivity” and “specificity.”

### IPL Activity Booklet

A four-page activity booklet was created using constructive alignment. Teaching-focused lecturers involved in the IPL activity design and delivery from two different disciplines. The workbook was co-created to allow students from both subjects to “lead” on content as per their specialist knowledge. This was intended to allow students to appraise their roles and apply integrative understanding of subject-specific content to peers from different backgrounds. This process was informed by Bloom’s Taxonomy principles [12].

**FIGURE 2:**
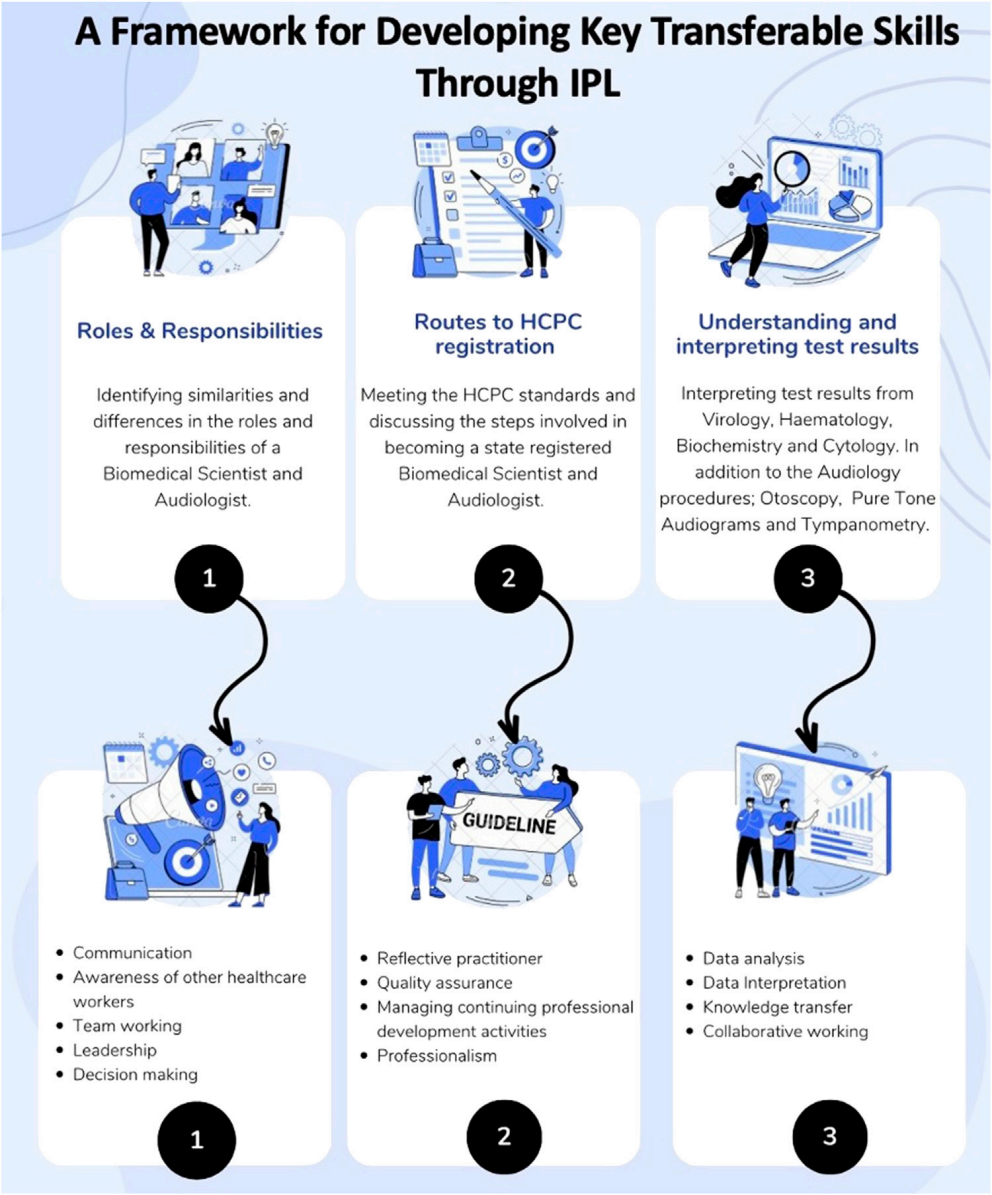
Details of the three IPL activities and the associated core transferable skills students developed.

The workbook was uploaded onto the VLE ahead of the workshop. Each activity was designed for students to identify and highlight commonalities and differences between the two professions. The booklet contained three tasks for the students to undertake. Activity 1 required students to work in mixed groups and assign key skills and roles that are typically attributed to either or both professions. Activity 2 required students to detail the key steps required to become an HCPC registered healthcare professional in the NHS. Activity 3 required students to interpret diagnostic data for both Audiology and Biomedical Science, with students explaining the purpose of each test to the other healthcare professional. The transferable skills gained through completing each activity are detailed in Figure 2.

### Data Collection and Analysis

Student responses following the IPL workshop were collected through a ten-item online questionnaire (Online Surveys, JISC, Bristol, United Kingdom). The survey was approved by the Health and Life Sciences Research Ethics Committee (Project #1494). Informed consent was built into the online survey prior to respondents accessing the survey. All responses to the survey were anonymous and participation was voluntary. The survey was advertised post-workshop using the VLE and remained open for 1 week.

Open and closed questions were included as part of the survey design and results were analysed both quantitively and qualitatively. To collate participants’ views of the IPL workshop and IPE, a five-point Likert scale was used to collate responses, which ranged from “strongly agree” to “strongly disagree.”

The Likert-scale responses were converted into a numerical format. Responses between the two student cohorts (Biomedical Science and Audiology) were compared using Mann-Whitney *U* tests. A non-parametric test was chosen as the data is ordinal and assumed to not have a normal distribution. All statistical analysis was performed using GraphPad Prism version 8.0.2 (GraphPad Software, United States). Statistical significance was determined by *p* < 0.05.

Free text responses allowed students to elaborate as to what they taught other students and what they learned from other students. Furthermore, free-text responses built an understanding of the student’s experiences of IPL and were analysed using thematic analysis [13]. The data was read multiple times by the first author to identify initial themes and this was repeated by all authors for triangulation, prior to a coding framework being developed and applied to the data set. The final themes were then agreed upon collectively.

## Results

A total of 108 students who were enrolled on first year Audiology and final year Biomedical Science degrees at Aston University participated in the workshop and completed the survey.

### Interactive Polling and Chat Function

During the workshop, interactive polling was deployed using a tool on Blackboard Collaborate, and on average there was ∼81% engagement across the three questions. The responses were anonymous, therefore the numbers responding from each programme could not be identified. Question 1 focussed on HCPC registration for both courses and 85% of students engaged with the poll. Question 2 focussed upon BMS pathology testing for viruses, highlighting the steps involved in PCR testing (engagement 88%) and question 3 was centred around audiology terminology (engagement 70%). Furthermore, students were able to use the chat function and as well as their microphone to both ask questions and answer questions.

### Response to Post Workshop Survey

**FIGURE 3:**
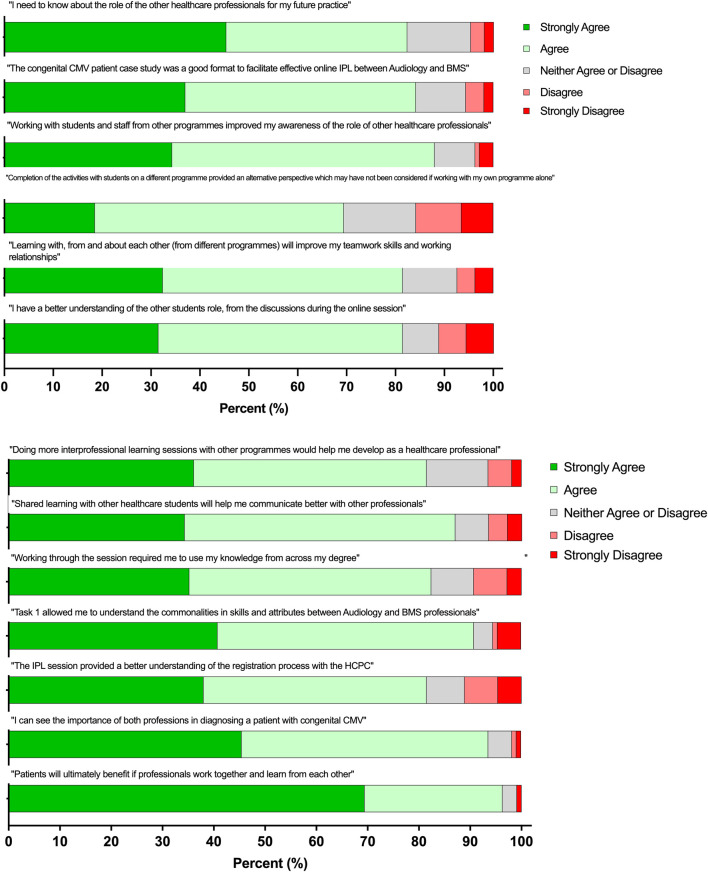
Student survey self-reported responses to the online IPL workshop (*n* = 108). A five-point Likert scale was used to answer each statement, with 1 = strongly disagree and 5 = strongly agree ± the standard deviation (SD). A Mann-Whitney U test was used to determine statistical significance (*p < 0.05) between the two student cohorts.

Participants responded to a series of statements relating to IPE and its role in the healthcare setting (Figure 3). Overall, a positive response was received from both cohorts, with 84.61% of students stating that they agreed or strongly agreed with all the statements. A Mann-Whitney *U* test was used to determine statistical significance (*p* < 0.05) and no significant differences were observed in the responses between the two student cohorts in response to any of the survey statements.

Over 82.4% of respondents “Agreed” or “Strongly agreed” that they need to know about the roles of other healthcare professions for their future careers, whilst 84.2% of respondents “Agreed” or “Strongly agreed” that the CMV congenital patient case study was an effective format to facilitate effective IPL (statements 1 and 2). A total of 88% of respondents stated that working with students and staff from other programmes improved their awareness of the role of other healthcare professionals (statement 3). A total of 69.4% of respondents reported that completing activities with students enrolled on the other healthcare programme provided an alternative perspective which they may not have considered outside of the IPL workshop (statement 4). Over 81.5% of respondents stated that the IPL workshop improved their communication, teamwork and working relationships, in addition to both cohorts learning with, from and about each other (statements 5, 6 and 8). Over 81.5% of respondents reported that the online workshop encouraged the application of subject specific knowledge, identified commonalities between the two professions, improved understanding of professional registration and recognised the value of both a Biomedical Scientist and an Audiologist in the diagnosis of a patient with CMV (statements 9, 10, 11 and 12). Finally, 96.3% of respondents “Agreed” or “Strongly agreed” that patients ultimately benefit from interprofessional working and 81.5% of respondents would welcome further opportunities to undertake IPL activities (statements 7 and 13).

### Free Text Responses for Thematic Analysis

**FIGURE 4:**
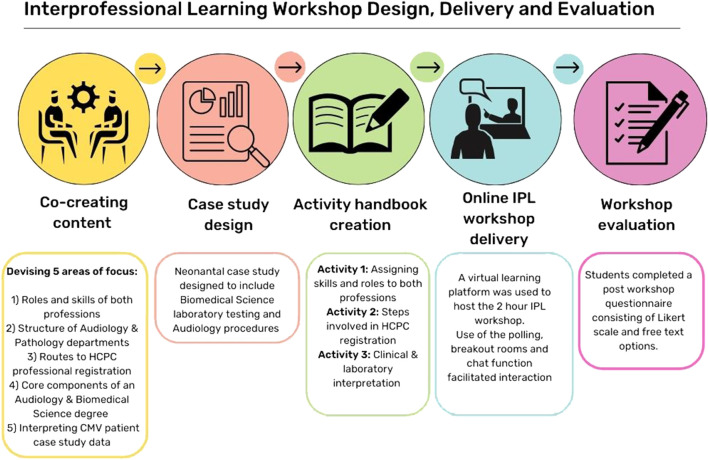
A visual depiction of the four primary themes derived from the thematic analysis conducted on the open-text responses obtained from Biomedical Science and Audiology students. The themes encompass what students felt they learnt and taught other students through the IPL workshop. Several students open-text response contained more than one theme.

To gain further insights into the experiences of the students undertaking the workshop, thematic analysis was carried out on two open-text responses. For question 6 – “*What did you learn from the students on the other programme?*” and question 7 – “*What did you feel you taught your peers when sharing your ideas or experience?*” 99% of the students responded (*n* = 107). The answers to these two questions were combined and analysis was conducted and four major themes were identified 1) the role of the clinician in the patient pathway, 2) appreciation of shared roles and responsibilities, 3) recognition of similarities in HCPC registration pathways and 4) the importance of working together to provide effective patient care (Figure 4).

### Theme 1: Appreciation of Shared Duties and Roles

Respondents recognised similarities and differences in the roles and duties that each profession carries out. This included specific testing for diagnosis purposes, but also calibration and communication across disciplines. It was the first time these students had met and they reflected on the shared roles the two professions had.

Comments included:

“I recognise similarities in the roles of Biomedical Scientists and Audiologists and differences related to their specialities”

“I learnt a lot about how to become a registered audiologist and what their role requires”

“Both professions carry out calibration of equipment”

“Their role in healthcare and how some tasks overlap”

“I gained another perspective of diagnosing and treating patients”

“I feel like I helped them understand the importance of our role in the healthcare setting”

### Theme 2: Recognition of Similar Registration Pathways

Respondents reported having a better understanding of not only their own registration routes but that of the other profession. They also recognised the importance of registration, the purpose of CPD and enhancing practice and the similarities in course accreditation.

“That all healthcare professionals go through a similar pathway in terms of career progression from university onwards.”

“How our courses and professions are more similar than they are different in terms of requirements.”

“How closely related our standards are while being part of different programs.”

“Their registration pathway and the similarities of the two roles”

### Theme 3: Working Together for Holistic Patient

Respondents realised the importance of their work and how it directly benefits the patient. Whilst Biomedical Science students do not have face-to-face interactions, they were able to highlight the role they play in patient care. The authors of this study consciously chose a relevant pathology (CMV) to highlight the involvement of both professions in newborn screening processes in the NHS. Both cohorts of students reflected on their roles within healthcare systems (e.g., the NHS) and how they contribute to effective patient care.

“Learnt more about the audiology profession and how we can work with them as BMS for better patient treatment”

“Importance of IPL and healthcare professionals working together for the patients”

“I learnt how BMS can work together with audiologists to deliver effective patient care”

### Theme 4: The Role of Clinicians in the Patient Journey

Respondents stated they had a better appreciation for how their roles contributed towards multi-disciplinary working. Students were able to showcase their roles whilst also reflecting on the value of their professions in the diagnosis pathway. Communication was highlighted as a key skill in delivering patient care.

Comments included:

“I feel like I helped them understand the importance of our role in the healthcare setting”

“Their specific roles and contributions to society and how we all eventually can work as a team to help the lives of others.”

“The audiology pathway and how important multidisciplinary work is for diagnosis.”

“How the interactions between both BMS and audiology and work together to obtain a diagnosis”

“How various blood tests can help with the diagnosis even if it’s related to audiology”

“Importance of communication and teamwork”

**FIGURE 5:**
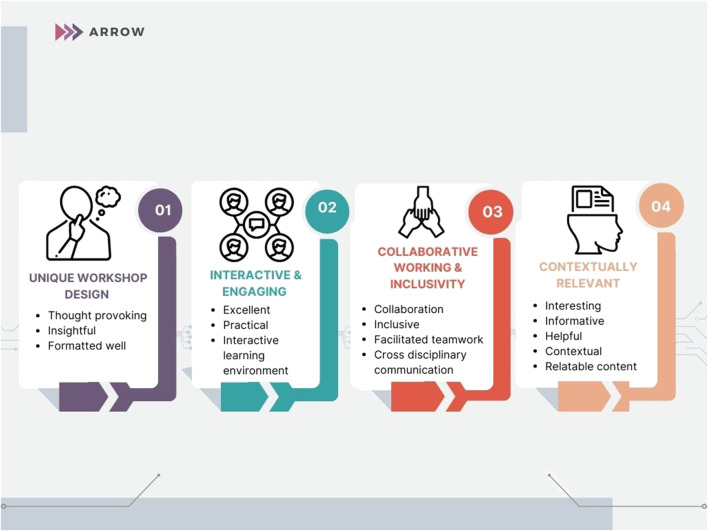
The four common themes identified through analysis of descriptive words to evaluate the workshop.

Students were also asked to provide three descriptive words to evaluate their session. Qualitative analysis included coding the data into four distinctive themes which include 1) Unique workshop design, 2) Collaborative working and inclusivity, 3) Interactive and engaging and 4) Contextually relevant (Figure 5).

## Discussion

### A Novel Approach to Bringing Biomedical Science and Audiology Together Through CMV

To our knowledge, this is the first study to evaluate the online delivery of IPL between two HCPC-approved programmes; Biomedical Science and Audiology. Both programmes need to meet the HCPC Standards of Education and Training, which require students to learn with and from other professions [5]. Students enrolled on healthcare degree programmes who partake in IPL opportunities are more likely to develop collaborative practice behaviours post-graduation in the workplace [14]. Thus, there is a growing significance in equipping healthcare professional students with skills that promote teamwork and cooperation [3, 15].

Biomedical Science is largely a non-patient facing profession, whilst Audiology professionals see patients/clients daily. Whereas both programmes include theoretical knowledge and clinical and/or practical skill components, it is important to provide opportunities relevant to real life contexts and to remind students that there is a patient behind each diagnosis. Therefore, the IPL workshop was designed for these two cohorts specifically to highlight the role that different healthcare professionals play in the patient journey. At face value it may seem that there is little overlap between these two professions, however, both play a key role in various conditions. CMV is one infection that can affect pregnant women and cause complications in neonates [16]. Collaboration between Biomedical Scientists and Audiologists assists in the diagnosis of conditions and improves hearing health in paediatric care. The use of a case study approach focused on CMV helps to facilitate sociocultural learning, which is deemed to be important for the delivery of effective IPL [17, 18].

### Workshop Delivery

Students in this study reflected upon the content of the workshop with 84.2% reporting that the CMV congenital patient case study facilitated effective online IPL between the two professions (Table 1). This is further supported by the thematic analysis shown in Figure 4, with students stating that they felt the CMV case study was contextually relevant, educational and informative. COVID-19 has reshaped the academic landscape and transformed the delivery of higher education online [19]. This innovative workshop was facilitated by IPL through the use of Blackboard Collaborate, a virtual learning environment for 108 students across two professions. Liaw et al. explored an online IPL activity through 3D simulation to develop and promote transferable skills across a range of healthcare cohorts, including Medicine, Nursing, Pharmacy, Physiotherapy and Occupational Therapy [4].

**TABLE 1:** Biomedical Science and Audiology student survey responses to the online IPL workshop (*n* = 108). A five-point Likert scale was used to answer each statement, with 1 = strongly disagree and 5 = strongly agree ± the standard deviation (SD). A two-tailed t-test was used to determine statistical significant (*p < 0.05) between the two student cohorts.

	Audiology Mean ± SD	Biomedical science Mean ± SD	*p*-value	Percentage “Agreed” and “Strongly agreed”
3.1. I need to know about the role of other healthcare professionals for my future practice	4.33 ± 0.98	4.19 ± 0.90	0.61	82.4
3.2 The congenital CMV patient case study was a good format to facilitate effective online IPL between Audiology and BMS	3.87 ± 0.83	4.18 ± 0.88	0.19	84.2
3.3. Working with students and staff from other programmes improved my awareness of the role of other healthcare professionals	4.27 ± 0.59	4.14 ± 0.87	0.48	88
3.4. Completion of the activities with students from a different programme provided an alternative perspective, which may not have been considered if working with own programme alone	3.80 ± 1.01	3.63 ± 1.10	0.57	69.4
3.5. Learning with, from and about each other (from different programmes) will improve my team work skills and working relationships	4.27 ± 0.70	3.99 ± 0.99	0.20	81.5
3.6. I have a better understanding of the other students role, from the discussions during the online session	4.07 ± 0.59	3.95 ± 1.12	0.54	81.5
3.7. Doing more interprofessional learning sessions with other programmes would help me develop as a healthcare professional	4.00 ± 0.76	4.11 ± 0.94	0.63	81.5
3.8. Shared learning with other healthcare students will help me communicate better with other professionals	4.07 ± 0.70	4.13 ± 0.92	0.76	87.1
3.9. Working through the session required me to use my knowledge from across my degree	4.27 ± 0.59	4.02 ± 1.02	0.20	82.4
3.10. Task 1 allowed me to understand the commonalities in skills and attributes between Audiology and BMS professionals	4.27 ± 0.70	4.20 ± 0.96	0.77	90.7
3.11. The IPL session provided a better understanding of the registration process with the HCPC	4.33 ± 0.82	3.99 ± 1.10	0.16	81.5
3.12. I can see the importance of both professions in diagnosing a patient with congenital CMV	4.33 ± 0.49	4.37 ± 0.73	0.83	93.5
3.13. Patients will ultimately benefit if professionals work together and learn from each other	4.60 ± 0.51	4.65 ± 0.65	0.76	96.3

### Roles and Responsibilities

Holistic patient care is dependent upon different healthcare professionals working together with an understanding of each other’s roles and remits. With the ever-changing healthcare landscape, the workforce must adapt to maintain high quality service delivery [14, 20]. Often within the healthcare setting an underappreciation of the roles of other professions can impact this [21], the results from the workshop show that 82.4% of students agreed that they need to know about the role of other healthcare professionals, with 88% stating that working with students from the other programme improved their awareness of other healthcare roles and responsibilities (Table 1). Thematic analysis also revealed that students have an appreciation of shared duties and roles (Figure 3), with students also recognising the similarities in the HCPC registration pathways and continual professional development [22]. Exposing students to the roles and responsibilities of different healthcare professionals whilst at the undergraduate level provides a foundation to build clinical relationships upon entering the healthcare setting. IPL has previously been shown to improve a student’s understanding of the skills and values between healthcare professions [23]. A greater appreciation of different roles can positively impact the functioning of teams and improve the standards of clinical practice and professionalism [21, 24].

### Teamwork and Communication

Another successful outcome of the workshop was that 81.5% of students recognised that participating in IPE will improve their teamwork skills and working relationships (Table 1). Previous work involving pharmacy, medical and nursing students have shown that IPL increased their knowledge of how to work effectively within a team, whilst increasing clarity around skillsets and limits of practice within a dynamic team [24–27]. Through the workshop 87.1% of students recognised that learning with other healthcare students will help to improve their communication skills when working with other professionals (Table 1). This is supported by previous research which has shown that IPL helps to foster effective communication [28, 29].

Students enrolled on healthcare degree programmes who partake in IPL opportunities are more likely to develop collaborative practice behaviours post-graduation in the workplace. Thus, there is a growing significance in equipping healthcare professional students with skills that promote teamwork and cooperation [3, 15]. Collaboration between Biomedical Scientists and Audiologists may assist in the diagnosis of conditions and improve hearing health in paediatric care. To promote an understanding of the role of a Biomedical Scientist in the patient pathway and the role of Audiologists in primary care, this IPL created a platform that allowed collaborative working.

The HCPC require professionals to be reflective practitioners and demonstrate the ability to reflect upon their own practice and skills. Through this study, students self-reported an increase in key transferable skills that they gained through the IPL activity, namely, communication, subject specific knowledge and teamwork. Final year Biomedical Science students have the opportunity to demonstrate these skills through the final year project poster presentation event; this takes place after the IPL workshop. Students need to communicate their final year scientific research project to peers and academics from different disciplines through the poster presentation event. When analysing the marks awarded for the poster presentation, the Biomedical Science student cohort achieved an average mark of 65% (2022–2023), compared to 61% in the previous academic year (2021–2022). Audiology students complete an additional IPL activity with pharmacy students in the second year where they utilise interdisciplinary and team working skills they have acquired from this IPL activity. These transferable skills are also put into practice during the clinical skills development module through peer group working across cohorts. Finally, these students undertake a final year clinical placement where they work with other healthcare professionals to support patient care.

## Future Work and Limitations

Whilst participants appreciated the comfort of learning from home during the workshop, digital inequalities and working environment considerations can disadvantage some students [19]. One unforeseeable drawback of hosting this workshop online is the occurrence of technical issues during its delivery, e.g., internet connectivity [3]. Additionally, during the online workshop, some students were reluctant to turn on their cameras; reasons included managing privacy, appearance considerations and home working environment, as evidenced in other literature [30, 31]. One challenge in the delivery of IPL is imbalances in cohort size between different programmes [32]. Whilst there was an Audiology to Biomedical Science student ratio of 1:6, this did not impact the overall success of the IPL session, with both student cohorts participating and interacting with one another during the workshop. Additionally, when examining the professional landscape, we find that there exists a notable ratio of approximately 23,000 Biomedical Scientists to 2,300 Audiologists in the United Kingdom [33, 34]. This translates to a proportional representation of one audiologist for every ten Biomedical Scientists in the workforce. This observation underscores the alignment of our educational setting with the existing workforce composition, reflecting our program’s adherence to the industry’s standards and demands.

In the case of Biomedical Science students, the workshop occurred during their final semester, just prior to their summer examinations and subsequent graduation. Consequently, it presents a challenge to procure longitudinal data regarding the sustained impact of the IPL activity, as these students have already concluded their studies at the university. Nevertheless, it is worth noting that a significant proportion of Biomedical Science graduates have opted to pursue postgraduate studies in fields such as medicine, physician associates, dentistry as well as various other patient facing healthcare-related programs. These advanced studies necessitate a demonstrable track record of engagement within multidisciplinary teams and a proficiency in interprofessional collaboration and many applicants used this IPL activity to evidence this. Furthermore, Audiology students graduate into patient facing roles and therefore work with Ear, Nose and Throat (ENT) specialists, GPs, Speech and Language Therapists, Physiotherapists and other services. Students experience this in their clinical placements across both NHS and private sectors. This experience therefore highlights the importance of working across specialisms which helps support student training in preparation for entering the healthcare workforce.

With regards to future improvements, some students suggested that they would welcome the opportunity to engage in IPL face-to-face. For example, one student’s feedback was that “*I think something like this would have worked nicely in person because it’s easier to discuss things and ask each other questions in person.*” However, there are logistical and timetabling challenges that universities face with ever growing numbers of students that may impede face to face delivery on campus. Furthermore, demographic data reveals that many students work part time and online delivery better accommodates their learning needs. This is further supported by the Higher Education Policy Institute (HEPI) student academic experience survey 2023, that reports that 55% of university students are now doing paid work and 76% of students reporting that the cost-of-living crisis has negatively impacted their studies [35].

IPL is acknowledged as beneficial for fostering collaboration amongst multidisciplinary teams and, ultimately, enhancing patient care [36]. Therefore, embedding IPL activities in year 1 and year 2 in the Biomedical Science curriculum would be advantageous as students would be developing important transferable skills that are required in their placement year with many of them working in the NHS setting. Furthermore, other work has reported that peer learning facilitates the understanding of both theoretical and practical concepts whilst developing student’s interpersonal and social skills [37]. Existing evidence has demonstrated peer learning to be beneficial for students belonging to marginalised groups. The majority of participants in our institution identify from minority backgrounds, thus supporting the need for further IPL opportunities.

## Conclusion

In conclusion, this novel collaboration between Biomedical Science and Audiology effectively delivered IPL and allowed both HCPC approved programmes to learn with, from and about each other. Students felt that the CMV case study was contextually relevant, informative and they strengthened their communication and teamworking skills. Whilst traditional IPL focuses on medicine and its associated programmes, IPL opportunities should be inclusive of other healthcare related Biomedical programmes. Healthcare departments recognise the importance of collaborative working to treat and diagnose patients and undergraduate allied healthcare degrees need to provide opportunities within their curriculum to foster this.

## Summary Table

### What Is Known About This Subject

• Biomedical Science and Audiology are both involved in diagnosing and managing patients with CMV.
• Both HCPC approved programmes require interprofessional learning (IPL) to be included in the curriculum.
• IPL seeks to promote knowledge exchange, skillset development and develop an appreciation of other healthcare professionals.

### What This Paper Adds

• A novel contribution to healthcare education through a contextually relevant patient condition.
• An IPL opportunity between a patient facing and a non-patient facing healthcare discipline.
• Students reported a greater appreciation and understanding of other healthcare professionals involved in patient care.

## Concluding Statement

This work represents an advance in biomedical science because it presents a novel IPL workshop with Audiology to strengthen transferable skills required in healthcare to support patient care.

## Data Availability

The raw data supporting the conclusion of this article will be made available by the authors, without undue reservation.
