# Supplementary material for: A Cytomegalovirus (CMV) Case Study to Promote Interprofessional Learning (IPL) Between Audiology and Biomedical Science Students in Higher Education
Source: Br J Biomed Sci. 2023 Nov 29;80:11680. doi: 10.3389/bjbs.2023.11680 (PMC10716233; doi:10.3389/bjbs.2023.11680)
Supplement: Supplementary file 1 [file DataSheet2.PDF]

Congential CMV: a novel virtual approach to promote inter-professional learning (IPL) between Biomedical science and Audiology in Higher Education

**BMS Student Names:**	
**Audiology Student Names:**	

Task 1 – Using the list of key skills/roles shown below, in your groups assign them under each professional title in the table on page 2. Note that some key/roles will be applicable to more than one professional title.

(10 minutes)

**Communicate with / advise the patient**	**Calibrate equipment before patient use**
**Interpret results**	**Conduct appropriate assessments**
**Recognise indications for further investigations**	**Supervise, mentor and support trainees**
**Maintain accurate records**	**Keep professional knowledge up to date, take responsibility for CPD**
**Measure glucose**	**Maintain and run specialist laboratory equipment**
**Accurately record data**	**Provide test results to other medical/healthcare colleagues**

Congential CMV: a novel virtual approach to promote inter-professional learning (IPL) between Biomedical science and Audiology in Higher Education

Role of a Biomedical Scientist	Role of an Audiologist

Congential CMV: a novel virtual approach to promote inter-professional learning (IPL) between Biomedical science and Audiology in Higher Education

Task 2 - Practising Biomedical Scientists and Audiologists need to be registered with their respective regulatory body in order to practise in the UK. In your groups provide a bullet point list of how to become a registered Biomedical Scientist and a registered Audiologist. Are there any similarities in the two processes? (15 minutes)

**How to become a registered Biomedical Scientist**	
**How to become a registered Audiologist**	

Task 3 – Interpreting results and understanding terminology (25 minutes)

Using the CMV case study, in your groups can you:

a) Interpret the blood test results given for the case study and describe their clinical significance

b) Briefly describe the purpose of the key audiology tests

Laboratory Parameter	Test Result e.g. raised, low etc.	Clinical Significance
**Haemoglobin (Hb)**		
**Mean Cell Volume (MCV)**		
**White Cell Count (WCC)**		
**Platelet Count (Plt)**		
**Serum Bilirubin**		
**Aspartate Transaminase (AST) & Alanine Transaminase (ALT)**		
**Audiology Test**	**Brief description of the purpose of the test**	
**Otoscopy**		
**Pure Tone Audiograms**		
**Tympanometry**		
