# Supplementary material for: A Cytomegalovirus (CMV) Case Study to Promote Interprofessional Learning (IPL) Between Audiology and Biomedical Science Students in Higher Education
Source: Br J Biomed Sci. 2023 Nov 29;80:11680. doi: 10.3389/bjbs.2023.11680 (PMC10716233; doi:10.3389/bjbs.2023.11680)
Supplement: Supplementary file 2 [file DataSheet1.PDF]

2023 IPL BMS and Audiology

Page 1: Participant Information Sheet (Student)

This study aims to evaluate the interprofessional education activity. The main benefit of the research is to improve awareness of your own academic programme and that of other programme(s), and have a better understanding how the programmes work together. In the clinical setting, collaboration between multidisciplinary teams is core to providing effective patient care. This IPL workshop was designed to allow collaboration between Biomedical Science and Audiology. This short survey will take no more than 5 minutes of your time and will assess your engagement with the IPL session.

Page 2: Consent

Project: Interdisciplinary Education at Aston

Please read the consent statements below. You need to tick yes to each statement to access the questionnaire. If you do not consent, please close the window and thank you for your time.

Once you have submitted your responses, we cannot remove your data as it is impossible to trace the identity of any respondant.

Thank you for your time, we appreciate your feedback.

1. I agree to take part in this study ✱ *Required*

○ Yes

Page 3: Survey questions

2. Please state the programme you are registered to ✱ *Required*

- ◯ Biomedical Sciences
- ◯ Audiology

3. Please tick the box for each statement ✱ *Required*

Please don't select more than 1 answer(s) per row.

Please select at least 12 answer(s).

	Strongly agree	Agree	Undecided	Disagree	Strongly disagree
Working with students and staff from other programmes improved my awareness of the role of other professionals	☐	☐	☐	☐	☐
I need to know about the role of other professionals for my future practice	☐	☐	☐	☐	☐
The virtual delivery of the IPL promoted interactivity and discussion of the tasks	☐	☐	☐	☐	☐

Discussion of the work stations with students from a different programme provided an alternative perspective (which may not have been considered if working with own programme alone)	☐	☐	☐	☐	☐
Learning with, from and about each other (from different programmes) will improve my team work skills and working relationships	☐	☐	☐	☐	☐
Patients will ultimately benefit if professionals work together and learn from each other	☐	☐	☐	☐	☐
I enjoyed working with students on the other programme on this activity	☐	☐	☐	☐	☐
I have a better understanding of the other students role, from the discussions during the online session	☐	☐	☐	☐	☐

Doing more interprofessional learning sessions with other programmes would help me develop as a healthcare professional	☐	☐	☐	☐	☐
Shared learning with other healthcare students will help me communicate better with other professionals	☐	☐	☐	☐	☐
Working through the session required me to use my knowledge from across my programme	☐	☐	☐	☐	☐
It was clear what I was required to do during the practical session on 28th February 2023	☐	☐	☐	☐	☐
I felt comfortable contributing to group discussions in the breakout rooms	☐	☐	☐	☐	☐

Task 1 allowed me to understand the commonalities in skills and attributes between Audiology and BMS professionals	☐	☐	☐	☐	☐
Did the IPL session provide a better understanding of the registration process with the HCPC?	☐	☐	☐	☐	☐
Was the congenital CMV patient case study a good format to facilitate effective IPL between Audiology and BMS?	☐	☐	☐	☐	☐
I can see the importance of both professions in diagnosing a patient with congenital CMV	☐	☐	☐	☐	☐

Page 4: video camera

4. **I feel comfortable interacting in university online learning sessions which require me to turn my video camera on** ✱ *Required*

- Strongly agree
- Agree
- Disagree
- Strongly Disagree

5. If you did not feel comfortable turning your camera on during the online session, what was the reason behind this? (please tick all that apply) ✱ *Required*

- Home environment being too noisy
- Family/house members interrupting
- Maintaining privacy of household members
- Poor internet connection
- Poor lighting
- Appearance related e.g hair/makeup
- Other

5.a. If you replied "other" please state reason

Page 5: Open questions and comments

6. What did you learn from the students on the other programme? ✱ *Required*

7. What did you feel you taught your peers when sharing your ideas or experience? ✱ *Required*

Please reflect on the activities you completed in the IPL and write down three descriptive words to describe the session:

8. Descriptive word 1:

9. Descriptive word 2:

10. Descriptive word 3:

11. Is there anything you can suggest to improve the session?

12. As a thank you for participating in this study, if you would like the opportunity to be entered into a prize draw to win a Love2Shop monetary voucher, please enter your email address below. The winner will be contacted in March 2023.

Page 6: Thank you

Thank you for taking time to complete this survey!
